# Absolute Quantification of *Grapevine Red Blotch Virus* in Grapevine Leaf and Petiole Tissues by Proteomics

**DOI:** 10.3389/fpls.2018.01735

**Published:** 2018-11-29

**Authors:** Natasha Buchs, Sophie Braga-Lagache, Anne-Christine Uldry, Justine Brodard, Christophe Debonneville, Jean-Sébastien Reynard, Manfred Heller

**Affiliations:** ^1^Proteomics and Mass Spectrometry Core Facility, Department for BioMedical Research (DBMR), University of Bern, Bern, Switzerland; ^2^Institute for Plant Production Science, Agroscope, Nyon, Switzerland; ^3^Bioreba AG, Reinach, Switzerland

**Keywords:** grapevine, *Grapevine red blotch virus*, proteomics, shotgun LC-MS/MS, parallel reaction monitoring, absolute quantification

## Abstract

Grapevine red blotch is a recently identified viral disease that was first recognized in the Napa Valley of California. Infected plants showed foliar symptoms similar to leafroll, another grapevine viral disease, on vines testing negative for known grapevine leafroll-associated virus. Later, the *Grapevine red blotch virus* (GRBV) was independently discovered in the US states of California and New York and was demonstrated to be the causal agent of red blotch disease. Due to its wide occurrence in the United States, vector transmission, and impacts on grape industry, this virus has the potential to cause serious economic losses. Despite numerous attempts, it has yet not been possible to isolate or visualize viral particles from GRBV-infected plants, thereby hampering the development of a serological assay that would facilitate GRBV detection in grapevine. In this work, mass spectrometry approaches were applied in order to quantify GRBV in infected plants and identify potential biomarkers for viral infection. We present for the first time the physical detection on the protein level of the two GRBV genes V1 (coat protein) and V2 in grapevine tissue lysates. The GRBV coat protein load in petioles was determined to be in the range of 100–900 million copies per milligram wet weight by using three heavy isotope labeled reference peptides as internal standards. In leaves on the other hand, the V1 copy number per unit wet tissue weight appeared to be about six times lower than in petioles, and about 300 times lower in terms of protein concentration in the extractable protein mass, albeit these estimations could only be made with one reference peptide detectable in leaf extracts. Moreover, we found in leaf and petiole extracts of GRBV-infected plants a consistent upregulation of several enzymes involved in flavonoid biosynthesis by label-free shotgun proteomics, indicating the activation of a defense mechanism against GRBV, a plant response already described for *Grapevine leafroll-associated virus* infection on the transcriptome level. Finally and importantly, we identified some other microorganisms belonging to the grapevine leaf microbiota, two bacterial species (*Novosphingobium* sp. *Rr 2-17* and *Methylobacterium*) and one virus, *Grapevine rupestris stem pitting-associated virus*.

## Introduction

Currently, more than 60 viruses have been reported to infect grapevine. One of the latest additions to this list is *Grapevine red blotch virus* (GRBV). GRBV infection has long been mixed up with grapevine leafroll disease due to similar foliar symptoms. When suspected leafroll-infected plants were tested negative for all grapevine leafroll-associated viruses, it was realized that another virus was at play (Calvi, 2011). Its genome was sequenced independently by three groups (Krenz et al., 2012; Al Rwahnih et al., 2013; Poojari et al., 2013), and was classified as a new member of the recently established *Grablovirus* genus in the *Geminiviridae* family (Varsani et al., 2017). GRBV is a circular single-stranded DNA virus of 3206 nucleotides with six predicted open-reading frames translated into three viral sense oriented proteins V1, V2, and V3, and three in the complimentary orientation (C1, C2, and C3), respectively (Sudarshana et al., 2015). While V1 has been identified as the coat protein and the complementary-sense gene products as replication-associated proteins, the exact function of V2 and V3 has been postulated as possibly related to the transport and localization of the virus within cells (Guo et al., 2015). However, none of these gene products have yet been experimentally confirmed on the protein level within grapevine cells, nor was it possible to visualize virus particles.

Grapevine red blotch virus is associated with the emerging red blotch disease in the United States (Al Rwahnih et al., 2013), and has been confirmed to be the causal agent of red blotch disease (Yepes et al., 2018). Typical symptoms caused by GRBV infection on red cultivars are reddening of leaf blade along the veins (Sudarshana et al., 2015). Fruit quality on diseased vines is negatively impacted compared to healthy controls. A very recent study discovered a reprogramming of the post-transcriptional machinery and abnormal transcription factor expression causing the attenuation of the normal berry ripening process (Blanco-Ulate et al., 2017). GRBV is widespread in North America (Krenz et al., 2014) while Europe seems to be GRBV free (Reynard et al., 2018a). There is evidence that wild grape is a carrier of GRBV and that it is transmitted by a treehopper *Spissistilus festinus* (Bahder et al., 2016; Perry et al., 2016).

Due to its wide occurrence as well as transmissibility and impacts on grape quality, this emerging virus has the potential to cause serious economic losses. Currently, polymerase chain reaction is used for diagnosis of GRBV infection of vines (Poojari et al., 2016; Reynard et al., 2018b). A serological test would be of great interest for the wine industry but attempts at developing it have failed so far. We therefore set out to implement a proteomics workflow, using nanoflow reversed phase liquid chromatography coupled to electrospray tandem mass spectrometry (nLC-MS/MS), in order to study (i) the grapevine leaf and petiole proteomes and GRBV-induced effects on them by a label-free shotgun approach and (ii) estimate the GRBV virus load on the protein level in grapevine tissue. Efficient protein extraction from recalcitrant plant tissues is an important first step toward these goals. Many protocols have been suggested in the past (Saravanan and Rose, 2004; Wang et al., 2006; Chatterjee et al., 2012; Palmieri et al., 2012), often using a precipitation step with trichloroacetic acid (TCA) alone or in combination with acetone, and extractions with ammonium acetate/methanol, or phenol, respectively. During the course of this work, we realized that such a protocol has to be adapted to each tissue type. Furthermore, the final protein extract has to be free of polymers and detergents in order to be amenable for proteolytic digestion and subsequent shotgun nLC-MS/MS analysis of peptides. With this technique, peptides are separated in chromatographic time on the nano-reversed phase column, and ionized directly when emitting from the column tip from where they enter the mass filters in the tandem mass spectrometer. Intact mass information of peptides and their corresponding peptide fragment fingerprints, together with intensities of all ions are recorded for peptide/protein identification and quantification (Zhu et al., 2010). This process is usually carried out in a data-dependent mode, whereby the software of the mass spectrometer decides which of the detected peptide ions are subjected to fragmentation and subsequent identification. In this operation mode, a mass spectrometer’s performance is rather unspecific and not very sensitive, resulting in a bias toward the more abundant proteins in a proteome, which can become an issue for plant leaf proteomics due to the immense concentration of the ribulose bisphosphate carboxylase protein (RuBisCo). Specificity and sensitivity can significantly be improved by applying a targeted approach, where the mass spectrometer restricts its acquisition time to a defined set of masses during a defined retention time window, blinding out the surrounding matrix. Common implementations are called selected reaction monitoring (SRM) or parallel reaction monitoring (PRM) with only one fragment ion, or the entire peptide fragment ion spectrum recorded, respectively (Gallien et al., 2012). We used the PRM approach in combination with three stable isotope labeled heavy peptides representing the GRBV coat protein as internal markers, also called AQUA peptides (Gerber et al., 2003), in order to determine the absolute concentration of GRBV coat protein in infected grapevine leaves and petioles.

## Materials and Methods

All chemicals and solvents were at least of analytical purity grade. The following products were purchased from Merck (Zug, Switzerland): acetone, ethylenediaminetetraacetic acid (EDTA), formic acid (FA), hydrochloric acid (HCl), methanol, TCA, and trifluoroacetic acid (TFA). The following products were from Sigma-Aldrich (Buchs, Switzerland): 2-mercapto ethanol, dithiothreitol (DTT), iodoacetamide (IAA), phenol solution, sodium dodecylsulfate (SDS), sucrose, *tris*(hydroxymethyl)-aminomethane (Tris), urea. LC–MS grade acetonitrile and ammonium acetate were from Fluka (Buchs, Switzerland), protease inhibitor cocktail from Roche (Rotkreuz, Switzerland), and sequencing grade endoproteases LysC and trypsin from Promega (Dübendorf, Switzerland).

### Grapevine Plant Material Collection and Storage

Plant reference samples were collected from the Agroscope grapevine virus collection (Switzerland). Five young and five mature leaves were collected and treated as individual samples from the top (young) and the lower part (mature) of the plants in October from non-infected plant 9021 and GRBV-infected plant 9034. Furthermore, a set of grapevine leaves and petioles were collected from the lower part (mature) of field cultivated non-infected Gamay plants 9106, 9107, 9108, and GRBV-infected plants 9115, 9116, 9117 during late spring/early summer. The leaf or petiole tissues from the same plant were pooled into one leaf or petiole sample, respectively. During the course of this study, *Grapevine rupestris stem pitting-associated virus* (GRSPaV) was diagnosed on plants 9106–9108 and 9115–9117. GRSPaV is a wide-spread virus in grapevine and it is commonly accepted to be a benign virus causing asymptomatic infections. All plant material samples were cut into smaller pieces, immediately frozen at −80°C, and shipped within several days on dry ice to the proteomics facility. The samples were stored at −80°C until grinding into a fine powder under liquid nitrogen in a RETSCH planetary ball mill PM-100 (Retsch GmbH, Haan, Germany). The powder material was stored again at −80°C until further use.

### Protein Extraction From Grapevine Leaves and Petioles

We have tested several solubilization protocols for their efficacy in protein yield, assessed by SDS–PAGE and Coomassie blue staining, bicinchoninic acid assay (BCA, Thermo Scientific, Rockford, IL, United States) or Bradford protein assay (Sigma-Aldrich, Buchs, Switzerland). The protocols tested were protein extraction with a hypotonic 50 mM Tris buffer, a urea buffer protocol as suggested by Palmieri et al. (2012), a direct TCA/acetone extraction procedure (Chatterjee et al., 2012), a two-stage hypertonic sucrose buffer solubilization without and with 2% SDS followed by phenol protein extraction and protein precipitation with ammonium acetate, different variations of TCA/acetone precipitation protocols (Saravanan and Rose, 2004; Wang et al., 2006), and a SDS-based buffer (personal communication with Dr. S Echevarria-Zomeño, Plant Biotechnology, ETH Zürich, Switzerland). None of these protocols lead to satisfactory extraction of proteins in both leaf and petiole tissue, either because of protein smears on SDS–PAGE, very poor protein yields, or non-reproducible protein yields from different preparations of the same input material. Satisfactory extraction for leaf tissue was achieved by the simple SDS extraction protocol, whereas for petiole tissue it was with the TCA/acetone precipitation protocol published by Wang et al. (2006).

#### Grapevine Leaf Sample Preparation for Shotgun nLC-MS/MS

For 50 mg of leaf powder, 0.2 mL of 50 mM Tris/HCl pH 8.0/4% (w/w) SDS/50 mM DTT/protease inhibitor cocktail (Roche, Rotkreuz, Switzerland) was added. The mixture was vortexed well with subsequent agitation for 30 min at 4°C. The resulting suspension was centrifuged for 15 min at 16,000 × *g* and 4°C. The supernatant was either used directly for SDS–PAGE or transferred to another reaction vial, heated for 5 min at 95°C, cooled to room temperature before addition of one-fourth volume of 1 M iodoacetamide in 100 mM Tris/HCl pH 8.0 and incubation for 30 min at 37°C. Proteins were precipitated by addition of 10% (w/w) TCA, 0.07% DTT (w/w) in acetone by overnight incubation at −20°C. Proteins were spun down for 15 min at 16,000 × *g* and 4°C. The supernatant was discarded and the pellet washed four times with ice-cold 0.07% (w/w) DTT in acetone. The final pellet was dried in ambient air and resuspended in 8 M urea/50 mM Tris/HCl pH 8.0. The protein concentration was assessed by BCA assay or comparing the Coomassie blue staining intensity with the one from 0.01 and 0.02 mg HEK293 cell lysate on a 12.5% (T) SDS–PAGE. This latter step was deemed necessary, as different attempts of protein assays resulted in inconsistent values compared with the SDS–PAGE staining intensities. The same problem with protein assays occurred for petiole protein extracts.

#### Grapevine Petiole Sample Preparation for Shotgun nLC-MS/MS

Petiole powder (100 mg) was weighed into a 2-mL reaction vial and filled up with 10% (w/w) TCA, 0.07% DTT (w/w) in acetone. The suspension was vortexed well for 30 s and centrifuged for 3 min at 16,000 × *g* and 4°C. The supernatant was removed carefully, and then the tube re-filled with 80% (v/v) methanol/0.1 M ammonium acetate followed by mixing and centrifugation as above. The supernatant was removed and the tube was filled once more with 80% (v/v) acetone/0.07 M DTT repeating the mixing and centrifugation steps. After removal of the supernatant, the pellet was dried from residual acetone in ambient air and dissolved in 0.5 mL 30% sucrose/2% SDS/5% 2-mercaptoethanol in 0.1 M Tris/HCl pH 8.0. An equal aliquot of 0.5 mL water saturated phenol (using 1 mM EDTA in 10 mM Tris/HCl pH 8.0) was added and mixed for 5 min at room temperature. After centrifugation for 3 min at 16,000 × *g* and 20°C, the upper phenol phase was carefully transferred into a fresh reaction vial without disturbing the white interface zone. Proteins were finally pelleted by filling the vial with 80% (v/v) methanol/0.1 M ammonium acetate and over-night incubation at −20°C, followed by centrifugation for 5 min at 16,000 × *g* and 4°C. The supernatant was carefully removed and the white pellet washed once with pure methanol and once with 80% acetone by strong mixing and centrifugation as above. The final pellet was either resuspended in 0.02 mL SDS–PAGE sample buffer or 0.02 mL 8 M urea/50 mM Tris/HCl pH 8.0.

### Shotgun nLC-MS/MS and PRM of Grapevine Leaf and Petiole Protein Extracts

Proteins extracted as described above were reduced, alkylated, and digested as described elsewhere (Braga-Lagache et al., 2016). An aliquot corresponding to 0.01 mg of leaf protein was digested using the same two stage digestion protocol without prior reduction and alkylation of proteins as these steps were already performed during protein extraction. Proteins from gel pieces were in-gel digested as described elsewhere (Gunasekera et al., 2012).

Data-dependent acquisition was done on a Fusion LUMOS mass spectrometer coupled with a Dionex Ultimate 3000 nano-UPLC system (Thermo Fischer, Bremen, Germany). Protein digests were loaded onto a pre-column (PepMap C18, 5 μm, 100 Å, 300 μm × 5 mm) at a flow rate of 50 μL/min with loading solvent (0.05% TFA in water/acetonitrile 98:2). After loading, peptides were eluted in back flush mode onto the analytical nano-column (C18, 3 μm, 155 Å, 0.075 mm i.d. × 150 mm length, Nikkyo Technos, Tokyo, Japan) using an acetonitrile gradient of 5–40% solvent B (0.1% FA in water/acetonitrile 4,9:95) in 60 min at a flow rate of 400 nL/min. The column effluent was directly coupled to the mass spectrometer via a nanoflex electrospray source (Thermo Fischer, Bremen, Germany). Precursor ion scans were recorded in the Fourier transform (FT) detector with resolution of 120,000 (at *m*/*z* = 250), maximum injection time (mIT) of 50 ms, and an automatic gain control (AGC) setting of 4e5. High energy collision activated (HCD) fragment spectra were acquired parallel to the FT scan with a top speed fragment spectra acquisition method of the most intense precursor ions in the linear iontrap for a cycle time of 3 s at a mIT of 35 ms, AGC of 1e4, and exclusion from further fragmentation for 30 s, using a relative HCD energy of 30%.

For the targeted, absolute quantification of GRBV coat protein, we used the three proteotypic stable-isotope labeled peptides NDVSGGGRNDVER[^13^C_6_,^15^N_4_], IYLSAASASGHTFK[^13^C_6_,^15^N_2_], and AAFNIFQR[^13^C_6_,^15^N_4_] (Bachem AG, Bubendorf, Switzerland) with a PRM approach on a QExactive HF mass spectrometer coupled with an Easy-nLC 1000 (Thermo Fischer, Bremen, Germany) and the same column setup as described above. PRM acquisition was done with a scheduled inclusion list between 8 and 15 min for NDVSGGGRNDVER (3+ ion, *m*/*z* = 458.8834, and *m*/*z* = 462.21950 for the light and heavy form, respectively), between 25 and 33 min for IYLSAASASGHTFK (3+ ion, *m*/*z* = 484.9209, and *m*/*z* = 487.5923), and between 34 and 41 min for AAFNIFQR (2+ ion, *m*/*z* = 483.76143, and *m*/*z* = 488.76560). Resolution was set to 30,000 (at *m*/*z* = 250), AGC target to 2e5, mIT to 130 ms, and a relative HCD energy of 28%, respectively.

**FIGURE 1:**
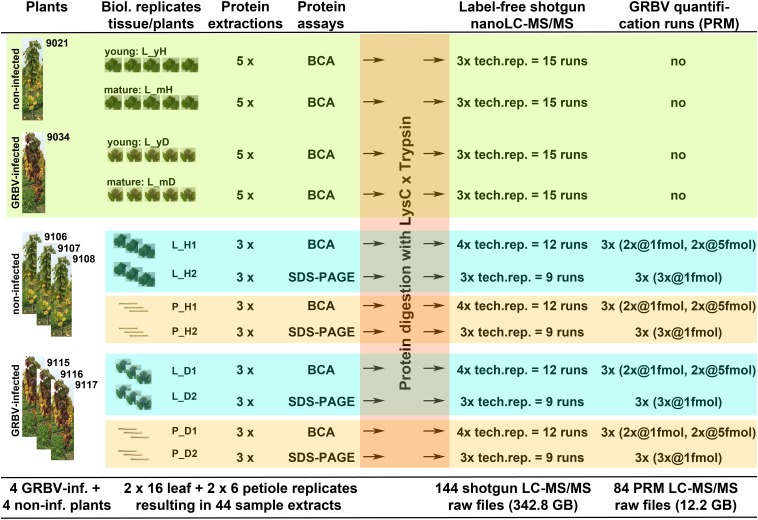
*Graphical outline of all proteome experiments described in this study. Light green section: protein discovery and study of variation within same plant* with plants 9021 (healthy) and 9034 (diseased); proteins from five individual leaves, called tissue replicates, were extracted from mature leaves, collected from lower part, and from young leaves, collected from top part of plants; each extract was run as three technical nLC-MS/MS replicates. *Blue-green and orange sections: GRBV quantification and protein extraction yield using different plants* with non-infected plants 9106, 9107, and 9108, as well as GRBV-infected plants 9115, 9116, and 9117; leaves and petioles were pooled tissue- and plant-wise; proteins from each of the 12 tissue samples were extracted and the protein content measured once by BCA and once by SDS–PAGE assay, respectively; again, each protein extract was analyzed three or four times by nLC-MS/MS for DDA, or targeted GRBV coat protein quantification by PRM assay. The header describes the different processing steps. The numbers from each processing step are summarized at the bottom of the cartoon.

Each protein extract digest from biological plant or tissue replicates was analyzed at least three times by nLC-MS/MS. Individual nLC-MS/MS runs were considered as technical replicates. An overview of the origin and name of each sample is sketched out in Figure 1.

### Data Processing and Bioinformatic Analysis

The DDA LC–MS/MS data were processed with MaxQuant software version 1.5.4.1 (Cox et al., 2014). The initial precursor mass deviation was set to 10 ppm and 0.4 Da for fragment peaks, respectively. Enzyme specificity was set to trypsin not allowing C-terminal cleavage after arginine and lysine when a proline was next in the sequence, and a maximum of three missed cleavages were allowed. Carbamidomethylation on cysteine was set as a fixed modification, methionine oxidation and protein N-terminal acetylation as variable modifications. The fragment spectra were interpreted with the Andromeda search engine against a forward and reversed protein sequence database containing all *Vitis vinifera* entries deposited at UniprotKB (version 2016_08), including other species with a *V. vinifera* association, and supplemented with the potential open-reading frames from GRBV (accession MF276895) and GRSPaV as sequenced at Agroscope (Reynard et al., 2018a), totaling in 65,423 proteins. Peptide spectrum matches and protein identifications were accepted at a 1% false discovery rate (FDR) and requiring at least two unique or razor peptides per protein group identification. For protein quantification, we relied on the MaxQuant built-in label-free quantification (LFQ) algorithm (Cox et al., 2014) and also applied a top3 peptide approach (Ahrné et al., 2013; Braga-Lagache et al., 2016). We also used the protein size normalized intensity-based absolute quantification (iBAQ) values for qualitative data comparisons (Ahrné et al., 2013). For top3, all peptide form identifications within a sample set in the evidence output file from MaxQuant were median normalized before imputation of missing values from the normal distribution of LOG2-transformed peptides using a down shift of 1.8 and a width of 0.3 standard deviations, a left-censored imputation strategy set as default in Perseus software (version 1.5.5.3) (Tyanova et al., 2016). Missing value imputation was carried out when there were at least two peptide form identifications in all technical replicates from the same plant tissue, otherwise the intensity was set to zero according to recommendations by Lazar et al. (2016). The three most intense peptide intensities were then summed to the protein group iTop3 intensity. The LFQ values were also LOG2-transformed, and missing values imputed with the same strategy as described above. Student’s two-sample *t*-tests were used to assess statistical significance of differentially expressed protein abundances using a 1% permutation-based FDR (*q*-values) correction for multiple hypothesis testing with Perseus software. To increase the protein annotation coverage, annotations from the first six proteins of each protein group were considered to replace the term “uncharacterized protein” where possible.

Parallel reaction monitoring data were processed with Skyline software 3.7.0.10940 (MacLean et al., 2010), extracting the five most intense fragment ion intensities (transitions) for AAFNIFQR peptide (y7, y6, y5, y4, y3), the eight most intense fragments for NDVSGGGRNDVER peptide (y5, y4, y3 charged 1+, and y12, y11, y10, y9, y8 charged 2+), and y4–y11 ions for the IYLSAASASGHTFK peptide, respectively. The raw intensity ratios were used without any normalization for the quantification of the native virus protein.

All mass spectrometry data are available via ProteomeXchange (identifier PXD011002) and https://panoramaweb.org/DyzAoQ.url.

## Results

### Protein Extraction Yields From Plant Tissues

**FIGURE 2:**
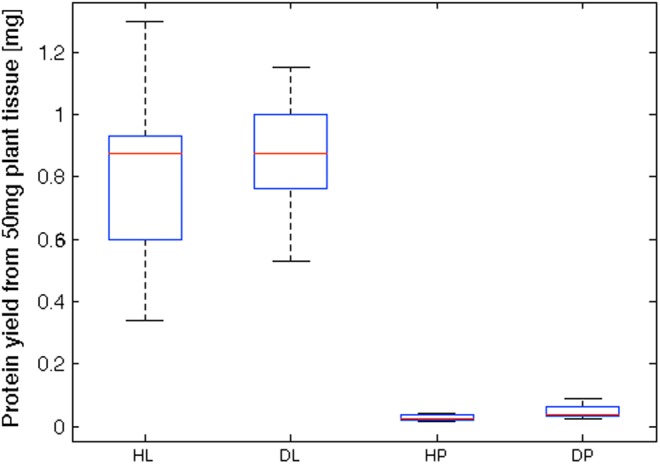
Boxplot representation of protein yields from grapevine tissue extracts. Calculated protein yields from 50 mg tissue powder extracted from leaves (L, *N* = 16) or petioles (P, *N* = 6). Extracts from GRBV-infected tissues (D) tended to yield higher protein amounts than from healthy (H) plants. Coefficients of variation were 29.8% for HL, 20.6% for DL, 34.9% for HP, and 48.8% for DP. The red line within each box delineates the median value, the bottom and top edge of each box the 25th and 75th percentiles, and the whiskers extend to the most extreme data points.

We evaluated different protocols for protein extraction from leaf and petiole tissue and concluded that a simple SDS extraction worked best for leaves, while the more complicated TCA/acetone protocol as described by Wang et al. (2006) was preferable for petioles. Our conclusions were based on reproducible protein patterns with distinct protein bands on SDS–PAGE and highest protein yields as determined by BCA assay. Calculated protein yields and coefficients of variations are summarized in Figure 2. Protein yields from GRBV-infected tissues tended to be higher albeit without statistical significance (Student’s *T*-test *p*-values of 0.36 and 0.11 for leaves and petioles, respectively). We also observed rather big variances with protein yields between extracts from different leaves and petioles (coefficient of variance between 20–30% and 35–50%, respectively). Based on median protein yields, we calculated a 36.9 times higher protein yield in leaves compared to petioles from healthy, and 23.6 times from GRBV-infected tissues, respectively.

### Shotgun Proteomics of Plant Tissue Protein Extracts

**FIGURE 3:**
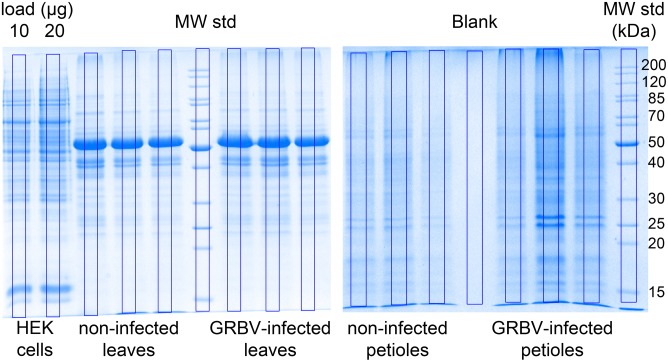
Coomassie blue stained gels of leaf (left) and petiole extracts (right). Equal volumes of final petiole or leaf extracts from three different plants were loaded on gel. The three replicates correspond, from left to right, to plants 9106, 9107, and 9108 (non-infected), and 9115, 9116, 9117 (GRBV-infected), respectively. The blue frames laid on top of each sample lane represent the regions used for calculating the total protein amount based on staining intensity and comparison with a 10 μg and 20 μg HEK293 cell lysate loaded on the very left. The molecular weight in kDa of the protein standards is given on the right (not marked are the 150, 100, and 60 kDa bands). From similar gels, we cut the region below 30 kDa molecular weight standard (MW Std.) and sliced it into seven equal fractions followed by in-gel digestion nLC-MS/MS in order to identify GRBV proteins.

We have processed in total 44 plant tissue extracts for this quantitative proteome study (Figure 1). First a batch of 20 leaf extracts was used to discover GRBV proteins and to define the proteome stability between leaves from the same plant at different maturation age. These 20 extracts were made of five different leaves from the following sources: (i) young, non-GRBV-infected leaves collected from the upper part of a GRBV-free plant, designated sample set L_yH (plant number 9021), (ii) mature leaves from the lower part of the same plant, sample set L_mH, as well as (iii) young (L_yD) and (iv) mature leaves (L_mD) from one GRBV-infected plant (number 9034) (Figure 1). Subsequently, we used plant-specific pools of leaves and leaf petioles from three different GRBV-free plants (9106, 9107, and 9108) and GRBV-infected plants (9115, 9116, and 9117) collected in early summer. Each of these pools was processed twice, albeit by the use of a different protein assay (see below). The sample sets were designated with the abbreviations L_H1 and L_H2 for non-infected leaves; replicate one and two and correspondingly L_D1 and L_D2 for infected leaves; and P_H1, P_H2, P_D1, and P_D2 for non-infected and infected petioles, respectively (Figures 1, 3). With the exception of the young leaves from plant 9034, all leaves from GRBV-infected plants showed the typical signs of GRBV infection.

**FIGURE 4:**
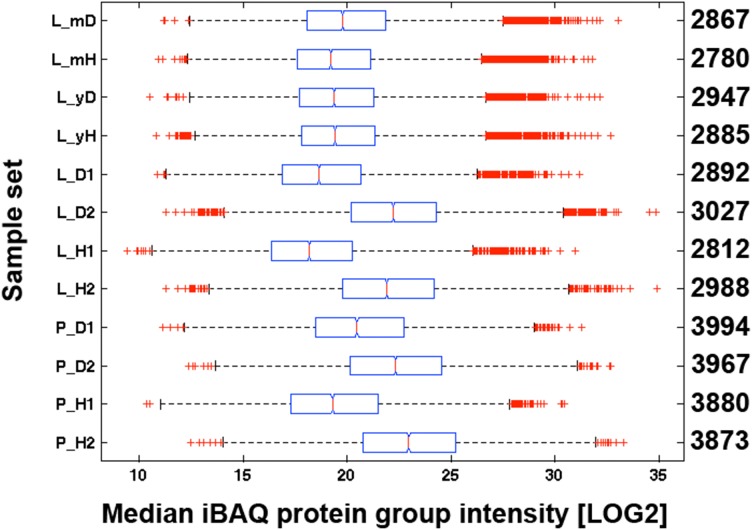
Median iBAQ protein group intensities of all replicates. iBAQ intensities of all replicates in each sample set (*y*-axis) are represented as boxplots. See Figure 1 for further explanations about the individual sample sets. The numbers on the right side *y*-axis represent the numbers of quantified proteins with an iBAQ intensity. With the exception of the young leaves, the difference between D and H was highly significant in each sample set according to a Kruskal–Wallis rank sum test (*p* < 0.01). The red line within each box delineates the median value, the left and right edge of each box the 25th and 75th percentiles, the whiskers extend to the most extreme data points not considered to be outliers, and outliers are represented individually with a red cross.

We loaded a theoretical amount of 500 ng protein digest with each shotgun nLC-MS/MS analysis based on BCA assay of the first replicates of leaf (L_H1, L_D1) and petiole pools (P_H1, P_D1). In order to gauge the accuracy of the protein load by BCA assay, we compared the Coomassie staining intensity of purportedly 20 μg proteins as measured by BCA from plant tissue extracts with 10 and 20 μg of a human embryo kidney cell line (HEK923) protein extract (Figure 3). We detected a weaker Coomassie staining intensity with plant tissue extracts compared to HEK923 and concluded that the BCA assay overestimated protein concentrations in plant tissue extracts. This is most likely due to interference of non-protein plant tissue compounds, such as chlorophyll, that were not completely removed during washing of protein precipitates. We therefore estimated the true protein concentrations using the second replicate sets of plant tissue pool extracts by comparing the overall Coomassie staining intensity of a square area of identical volume laid across the center of each lane with the HEK923 intensities after correction with the blank lane (Figure 3). The molecular weight standards served as internal controls to normalize protein quantification between gels. Within each sample set, we could quantify consistently more proteins in GRBV-infected plant tissues than in non-infected (Figure 4). Moreover, the median MaxQuant iBAQ protein intensities in infected plant tissues were significantly higher, with the exception of young leaves from the same plant and the second petiole replicate with corrected protein loads (Figure 4). This observation led us to conclude that GRBV infection alters the tissue rigidity, allowing to extract more protein. This is corroborated by the increase in iBAQ intensities between mature leaves showing signs of viral infection, and young infected leaves without any signs yet (L_mD vs. L_yD), while the opposite was true for the non-infected leaves (L_mH vs. L_yH, Figure 4).

#### Protein Identification Summary

We identified and quantified in total 4707 protein groups by the iTop3 quantification approach. The leaf proteome sets (two replicates of pooled leaves from six different plants, and five individual young, respectively, mature leaves from two plants, i.e., L_H1, L_H2, L_D1, L_D2, L_yH, L_mH, L_yD, and L_mD) encompassed 3676 quantifiable protein groups with 2305 identified in all sets (62.7%). In the two petiole replicates (P_H1, P_H2, P_D1, P_D2), we quantified 4195 protein groups with 1119 being unique to petioles. A total of 2026 protein groups were identified in all datasets (Supplementary Table and Supplementary Figure 1).

Of particular interest was the comparison of leave proteomes between leaves collected from the same plant. For this, we subjected the iTop3 intensities of the 1483 protein groups identified in all 20 leaf extracts (L_yH, L_yD, L_mH, and L_mD) to a principle component analysis (PCA) (Supplementary Figure 2). The PCA plot revealed two things: First, the extractable proteomes of mature leaves were very similar between samples, but not between young leaves, even though the leaves were from the same plant. Second, GRBV infection clearly changes the proteome in mature leaves. Including pooled tissue from different plants into the PCA revealed a similar spread as for different leaves from the same plant in the first component but remarkably less in the second component (Supplementary Figure 3).

**FIGURE 5:**
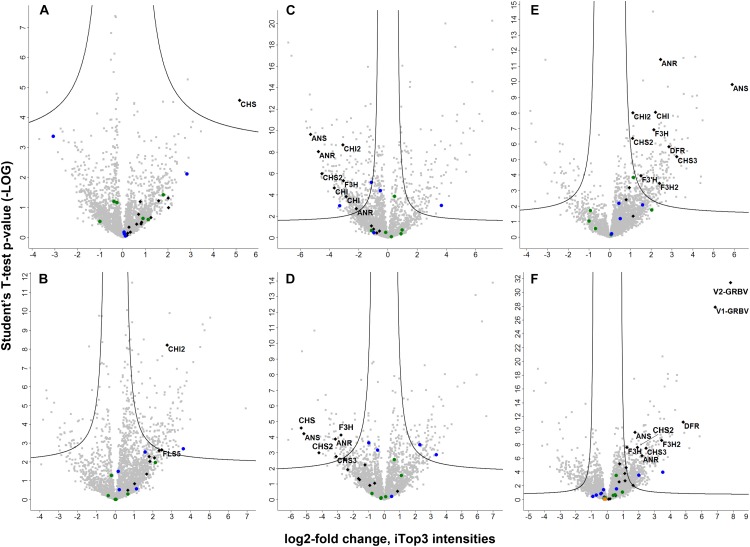
Volcano plots representing the statistical test results of quantitative label-free shotgun proteome results. Student’s *t*-test *p*-values (as -LOG) from the different tests performed are given on the *y*-axis with the corresponding LOG2-fold changes of iTop3 intensities on the *x*-axis, respectively. Black diamonds represent flavonoid biosynthesis enzymes and the two GRBV proteins, with latter only identified in petioles. The gene names of differentially expressed proteins are given next to their corresponding diamonds. The ORF5 protein of GRPaV, also quantified only in petioles, is indicated by an orange square in panel F. The filled circles represent proteins from the two identified bacterial species, *Methylobacterium* in blue and *Novosphingobium* in green. The following test results are shown in the six panels: **A**, GRBV-infected (L_yD) vs. non-infected young leaves (L_yH); **B**, GRBV-infected (L_mD) vs. non-infected mature leaves (L_mH); **C**, mature (L_mH) against young non-infected leaves (L_yH) from the same plant; **D**, mature (L_mD) against young GRBV-infected leaves (L_yD) from the same plant; **E**, GRBV-infected (L_D1 and L_D2) against non-infected leaves (L_H1 and L_H2) from three different plants; and **F**, GRBV-infected (P_D1 and P_D2) against non-infected petioles (P_H1 and PH2) from three different plants. If the LOG2-fold change is on the positive side, the corresponding protein was more abundant in the first mentioned sample set, if negative it was more abundant in the second sample set, respectively.

Most grapevine protein sequences in UniprotKB are currently not yet reviewed, with 54,424 entries marked as machine annotated entries from TrEMBL and only 171 human reviewed in SwissProt. By using up to the first six proteins in each protein group, it was possible to putatively annotate 1135 protein groups. GRBV proteins V1 and V2 could be identified consistently only in petiole extracts of GRBV-infected plants (Table 1 and Figure 5, panel F). However, MaxQuant/Andromeda also identified one peptide (GVVLPTENVTDGLHDIYFWIILDR) with one single peptide spectrum match in the second nLC-MS/MS run of non-infected plant 9106 (Supplementary Figure 4). This peptide identification can be rejected as a false positive match due to the weak evidence for its identification. We also attempted to identify V1 and V2 in the low molecular weight region (<30 kDa) of SDS–PAGE separated leaf extracts, however without any success.

**Table 1:** Identified gene products of foreign taxonomies.

Taxonomy	Gene name	Protein description	L_yD-L_yH		L_mD-L_mH		L_D-L_H		P_D-P_H	
			*diff.*	*q*	*diff.*	*q*	*diff.*	*q*	*diff.*	*q*
GRBV	V1	V1 protein, potential							25.4	**0.000**
	V2	V2 protein, potential							26.4	**0.000**
GRSPaV	ORF5	28 kDa coat protein							−0.2	0.816
*Methylobacterium* sp. GXF4	ssuB	Aliphatic sulfonates import ATP-binding protein	2.8	0.108	1.6	0.020	1.6	0.022	−0.4	0.304
	adk	Adenylate kinase	−3.1	0.036	3.6	**0.006**	0.1	0.815	−0.2	0.454
	acsF	Aerobic magnesium-protoporphyrin IX monomethyl ester [oxidative] cyclase	0.1	0.922	0.2	0.543	0.5	0.247	−0.7	0.192
	dnaK	Chaperone protein	0.1	0.918	0.2	0.508	0.4	0.207	0.6	0.158
	rplB	50S ribosomal protein L2							−0.2	0.609
	WYO_3190	Putative silver efflux pump							2.0	***0.001***
	WYO_3159	Uncharacterized protein							3.5	**0.000**
	WYO_1571	GntR family transcriptional regulator							−0.9	0.191
	WYO_1103	Signal peptide peptidase SppA, 36K type	0.1	0.958	1.1	0.287				
*Novosphingobium* sp. Rr 2-17	nuoI	NADH-quinone oxidoreductase subunit I					−0.9	0.077	0.4	0.357
	hppA	K(+)-insensitive pyrophosphate-energized proton pump	−0.3	0.625	0.0	0.992	1.1	0.013	0.5	0.271
	msrA	Peptide methionine sulfoxide reductase	−0.4	0.533	−0.2	0.450	0.1	0.877	−0.1	0.738
	WSK_1030	Sensor histidine kinase	1.8	0.235	2.1	0.026	−0.7	0.338	0.6	0.126
	secA	Protein translocase subunit	0.9	0.535	−0.4	0.643	−20.8	***0.141***	1.0	0.089
	WSK_0704	Uncharacterized protein	−1.0	0.567	0.0	0.967	2.0	0.022		
	WSK_1281	Citrate lyase subunit beta	1.1	0.534	0.7	0.518			−0.2	0.649

*Median LOG2-fold changes of iTop3 intensities between GRBV-infected and non-infected plant tissues are given (diff.) together with the corresponding q-value (q) calculated after imputation of missing values as described in the text (Figure 5). We considered q-values of ≤0.01 as a threshold for significance. For test description in the column header, the sample descriptions as given in Figure 1 were used. Significantly changed protein expression is indicated by q-value numbers in bold when confirmed by LFQ expression levels. When there was significance achieved only by iTop3 and not confirmed with LFQ (or vice versa), the q-value numbers are given in cursive font. Note that for some proteins big absolute median differences (>19) without significance resulted because the protein was identified only in one out of several tissue extracts.*

In petioles, we could also identify the 28 kDa coat protein (ORF5 protein) from GRSPaV in GRBV-infected and non-infected plants (Table 1 and Figure 5, orange square of panel F). GRSPaV is a wide-spread virus in grapevine cultivars causing no pathological symptoms in grapevine plants. In addition, we identified in all datasets from all plants several proteins from the two bacteria *Novosphingobium* sp. *Rr 2-17* (10 proteins) and *Methylobacterium* sp. *GXF4* (11 proteins) (Table 1). Both bacterial genomes were sequenced in 2012 by the same group (Gan et al., 2012a,b). The genome for *Methylobacterium* sp. *GXF4* had been isolated from the xylem of grapevine plants, while the one from *Novosphingobium* sp. *Rr 2-17* was found in the crown gall tumor of grapevine plants, respectively. Our plants did not show any signs of crown gall tumor.

#### Statistical Evaluation of Differential Protein Expression

In order to determine changes in biological pathways induced by GRBV-infection, we statistically evaluated the protein abundances between GRBV-infected and non-infected plant tissues in each data set. We defined the statistical significance for differential expression by a *q*-value of ≤0.01 and a minimal LOG2-fold change of approximately 1. The *q*-values were determined by a permutation-based *p*-value distribution in order to correct for multiple hypothesis testing. We would like to stress here that the calculated *q*-values for proteins which were quantified only in one group (let’s call them on/off proteins) are hypothetical. However, such on/off proteins should be treated as significantly changed in their expression level, and not neglected for the statistical test. Based on the numbers of differentially expressed proteins, we could observe the following. The proteome analysis of five young leaves from the same plant in each group (L_yD against L_yH) resulted in a weaker discriminating power between GRBV infected and healthy plants than with mature leaves (L_mD against L_mH) (Figure 5, panel A compared to panel B). Consequently, only three proteins were significantly changed in expression between non-infected and GRBV-infected young leaves, with chalcone synthase (CHS) being upregulated in latter (Figure 5, panel A). In mature leaves of the same plants, 67.7% of proteins had a higher expression level in GRBV-infected plants and 150 had at least a twofold change in expression with a statistically significant *q*-value of ≤0.01 compared to only 38 with an increased expression in healthy tissue (Figure 5, panel B). This trend was confirmed by the analysis of leaves from different plants, where 71.1% of all proteins showed an increased expression level in GRBV-infected leaves with 215 being significant, compared to only 45 significantly increased proteins in healthy plants (Figure 5, panel E). In petioles, we also detected more proteins with a positive test difference between GRBV-infected and non-infected plants (59.1%) and 370 reaching significance compared to 153 of significantly lower expression (Figure 5, panel F). We also compared protein expression between mature and young leaves without or with GRBV infection, respectively (Figure 5, panels C and D). We observed a higher protein expression in young leaves with 66.1% of proteins having a higher intensity and consequently 291 proteins being significantly overexpressed in young tissue. This trend to higher protein expression in young compared to mature leaves was abrogated by GRBV-infection with a 49:51 ratio in test difference and around 80 proteins significantly up- or down-regulated between mature and young leaves (Figure 5, panel D). The same observations were made by using the LFQ protein intensities, albeit with lower numbers of observed differentially expressed proteins (Supplementary Figure 5).

We then used the PANTHER Classification System website tool^1^ in order to find potentially enriched biological pathways in either the up- or down-regulated protein groups in each dataset. We observed up-regulation of enzymes involved in the generation of precursor metabolites and energy in GRBV-infected tissues of mature leaves and petiole datasets. On the level of individual protein groups, Student’s *t*-tests identified anthocyanidin synthase (gene name ANS), also known as leucoanthocyanidin oxygenase (LDOX), anthocyanidin reductase (ANR), and chalcone synthases (CHSs) (in different isoforms CHS, CHS2, and CHS3) as proteins consistently upregulated in GRBV-infected plant tissues (Figure 5 and Table 2). ANS is the last enzyme in the synthesis of anthocyanin, a flavonoid compound (Wilmouth et al., 2002). CHSs are key enzymes in the first step of the biosynthesis of flavonoids, catalyzing the production of naringenin (Dao et al., 2011), while ANR transforms the products of ANS to pro-anthocyanidins (Gutha et al., 2010).

**Table 2:** Identified *Vitis vinifera* gene products involved in flavonoid biosynthesis according to Gutha et al. (2010).

Gene name	AC	Protein description	L_yD-L_yH		L_mD-L_mH		L_D-L_H		P_D-P_H	
			*diff.*	*q*	*diff.*	*q*	*diff.*	*q*	*diff.*	*q*
CHS	A2ICC5	Chalcone synthase	24.8	**0.008**			1.1	0.084	1.2	0.011
F3H	A2ICC8	Naringenin, 2-oxoglutarate 3-dioxygenase	0.8	0.420	1.0	0.199	2.1	**0.000**	1.2	**0.005**
ANS	A2ICC9	Leucoanthocyanidin dioxygenase (LDOX)	1.2	0.494	1.7	0.076	5.9	**0.000**	1.8	**0.000**
CHI	A5BMU2	Chalcone–flavonone isomerase family protein	0.3	0.852	2.3	***0.010***	2.2	**0.000**	0.7	0.080
	A5C8D5	Chalcone–flavonone isomerase family protein	0.2	0.934			0.8	0.068		
FLS5	A5BV11	Flavonol synthase	0.8	0.600	2.5	**0.010**	0.9	0.032		
CHI2	D7T475	Chalcone–flavonone isomerase 2	0.3	0.771	2.8	**0.000**	1.1	***0.003***	0.8	0.049
ANR	D7U6G6	Anthocyanidin reductase	0.2	0.891	1.8	0.028	2.4	**0.000**	2.2	**0.000**
	Q5FB34	Anthocyanidin reductase	0.8	0.636	1.8	0.022			23.5	0.011
F3H2	E3TBM5	Flavanone 3-hydroxylase 2 (Fragment)	21.3	0.514			2.4	**0.001**	3.5	**0.000**
DFR	P93799	Dihydroflavonol 4-reductase					2.9	**0.000**	4.8	**0.000**
F3′H	Q2UYU6	Flavonoid-3-hydroxylase	0.6	0.660	0.7	0.380	1.5	0.005	1.1	0.025
CHS2	Q8W3P5	Chalcone synthase	2.0	0.347	2.1	0.020	1.1	0.005	1.9	**0.000**
CHS3	Q8W3P6	Chalcone synthase	2.0	0.250			21.1	**0.000**	2.5	**0.000**
lar2	D7SIV3	Putative leucoanthocyanidin reductase 2							0.5	0.269
UFGT	D7T7R5	Anthocyanidin 3-*O*-glucosyltransferase 2							0.1	0.849
FLS4	F6H0T8	Flavonol synthase							0.2	0.722
MT	G0YKW8	Anthocyanin *O*-methyltransferase							−0.4	0.340
F3′5′H	Q2UYU7	Flavonoid-3,5-hydroxylase	1.6	0.294					1.1	0.018

*Median LOG2-fold changes of iTop3 intensities between GRBV-infected and non-infected plant tissues are given (diff.) together with the corresponding q-value (q) calculated after imputation of missing values as described in the text (Figure 5). We considered q-values of ≤0.01 as a threshold for significance. For test description in the column header, the sample descriptions as given in Figure 1 were used. Significantly changed protein expression is indicated by q-values in bold numbers when confirmed by LFQ expression levels. When there was significance achieved only by iTop3 and not confirmed with LFQ (or vice versa), the q-value numbers are given in cursive font. Note that for some proteins big median differences (>19) without significance resulted because the protein was identified only in one out of several tissue extracts.*

### Absolute Quantification of GRBV Coat Protein

**FIGURE 6:**
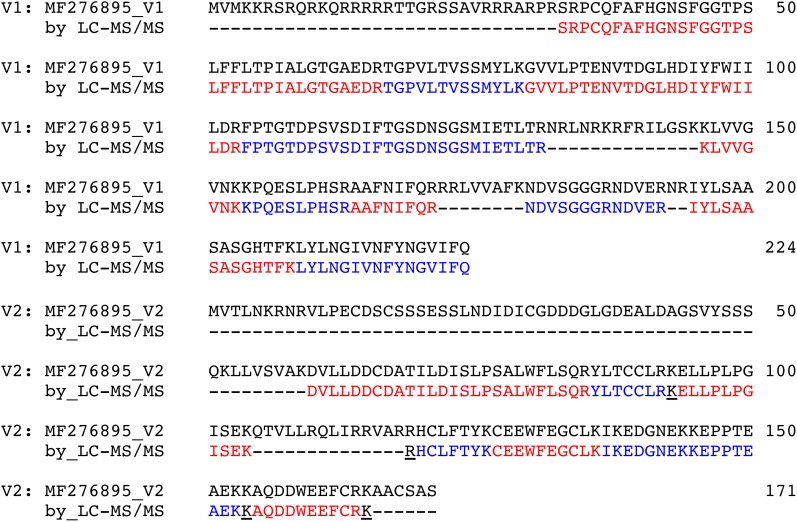
Sequence coverage of coat (V1) and V2 GRBV proteins by nLC-MS/MS. All peptides identified by in-gel digestion or shotgun LC-MS/MS are labeled in alternating red and blue color. The underlined lysine and arginine residues indicate missed-cleavage sites. Coat protein coverage was from 33 to 224 (75.0%), V2 from 60 to 165 (53.8%), respectively. The V1 and V2 sequences are given as deposited with accession MF276895 in the NCBI database.

As mentioned above, we also sequenced the proteins with an apparent molecular weight smaller than about 30 kDa by way of in-gel digestion from leaf and petiole extracts separated by SDS–PAGE. We could identify the V1 and V2 GRBV proteins in petioles of GRBV-infected plants but not in the leaves. V1 and V2 were the only GRBV proteins identified from the six possible open-reading frames of the GRBV genome as deposited in the NCBI GenBank database with accession number MF276895 (Reynard et al., 2018a). We achieved sequence coverages of 75.0% for the V1 (amino acid range of 33–324) and 53.8% for V2 (range 60–165) by combining the shotgun and in-gel results (Figure 6). The missing N-terminal protein parts were likely missed due to the fact that proteolysis with trypsin produces too short or too long peptides, not amenable to sequencing with our nLC-MS/MS setup. We focused our quantification efforts on the potential coat protein V1. For this, we chose the three peptides AAFNIFQR (A-R), NDVSGGGRNDVER (N-R), and IYLSAASASGHTFK (I-K) as surrogates for the V1 protein, based on the facts that they were consistently detected, had different retention times, and represented unique peptide sequences in the proteome under investigation. The third peptide I-K can furthermore be used to distinguish this GRBV strain from others deposited in public sequence databases where the C-terminal lysine is replaced by an arginine.

**FIGURE 7:**
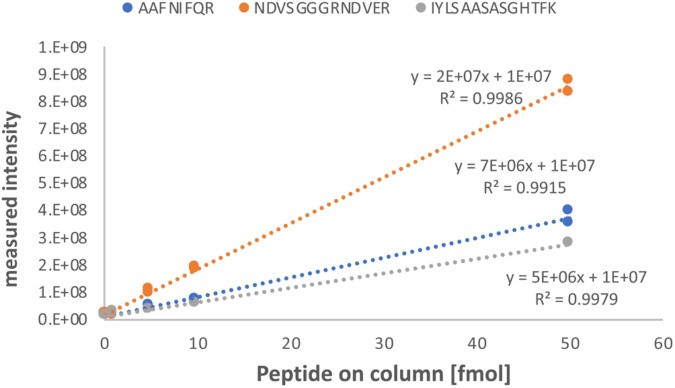
Calibration curve of AQUA peptides spiked into a combined protein extract from petioles of non-infected plants. Each of the three peptides (sequences and data label on top) were spiked into the combined petiole extract of non-infected plants 9106, 9107, and 9108, to achieve a peptide load on column of 0.1, 0.5, 1, 5, 10, and 50 fmol. Linear regression curves with the corresponding coefficient of determination (*R*^2^) are given. The *y*-axis represent the absolute signal intensity and the *x*-axis the peptide load on column in femtomole.

**FIGURE 8:**
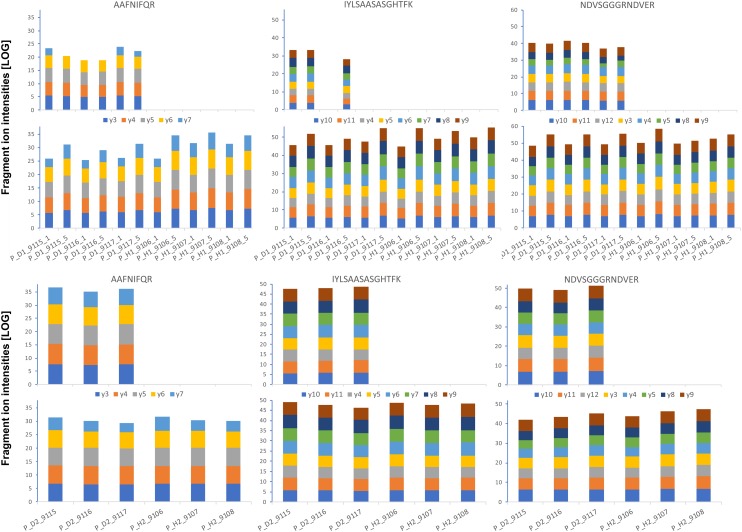
Absolute GRBV coat protein (V1) quantification by PRM assay in petiole extracts. The intensities for each fragment *y*-ion, represented with different colors as indicated between the upper and lower bar graphs, were extracted by Skyline software from the raw data for each of the three analyzed peptides (sequences indicated on top), and the values from the technical replicates were summed to 1 value per plant extract. The log-transformed intensities are illustrated in each bar graph. *Upper panel: Petiole extracts P_D1 and P_H1 from three different plants, each, analyzed with 1 and 5 fmol heavy-labeled AQUA peptide spike-in.* The AQUA peptide concentration is indicated by the number after the plant replicate annotation given on the *x*-axis of the lower bar graph, e.g., P_D1_9115_1 for first replicate extract from GRBV-infected plant 9115 with 1 fmol AQUA peptide. *Lower panel: The second replicate petiole extracts P_D2 and P_H2 analyzed only with 1 fmol AQUA concentration.* The measured intensities of the native peptides are shown in the upper bar graphs in each panel, and the spiked-in AQUA peptide intensities in the lower bar graphs, respectively.

**FIGURE 9:**
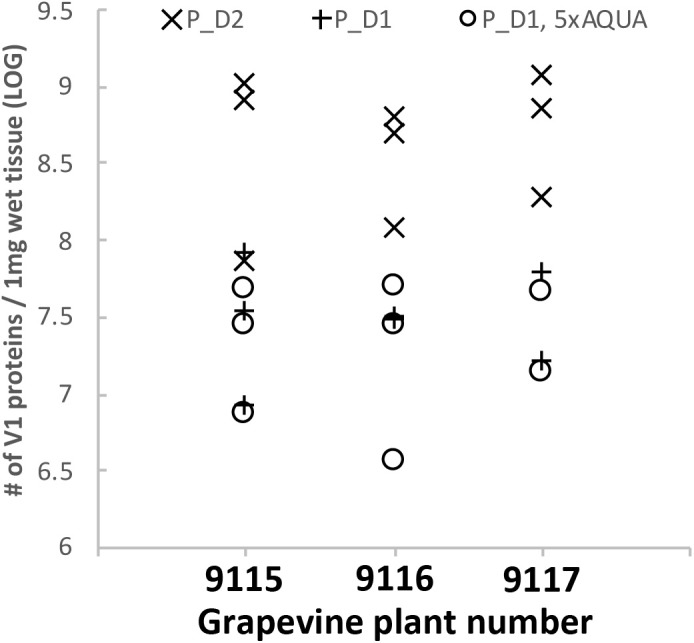
Absolute quantification of GRBV coat protein (V1) in grapevine petioles. The three heavily labeled AQUA peptides were spiked to each petiole extract (*x*-axis) at a concentration corresponding to 1 fmol (symbols × and +) or 5 fmol (°) on column, respectively. Petiole extracts of sample set P_D1 (+ and °) and P_D2 (×) were used. The three data points for each set represent the calculated number of V1 molecules per 1 mg wet tissue calculated from the three AQUA peptides. AQUA peptide IYLSAASASGHTFK showed consistently a lower response than the two other peptides. The *y*-axis represents the calculated number of V1 protein molecules extracted from 1 mg wet tissue in logarithmic scale.

**FIGURE 10:**
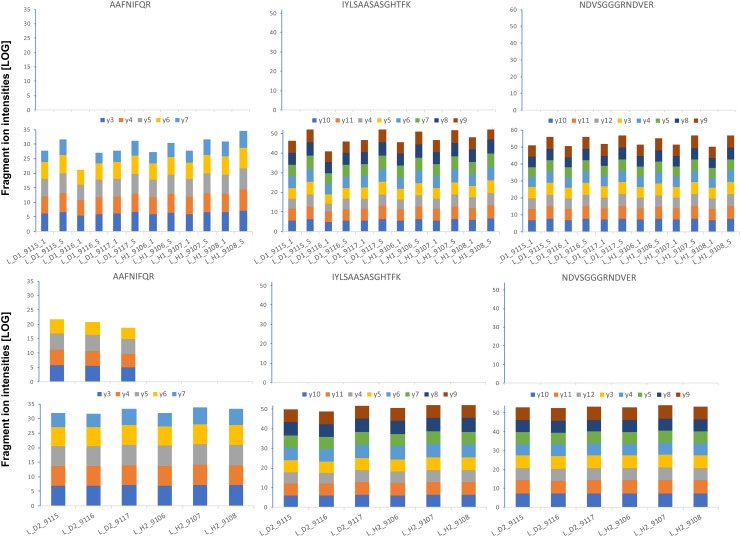
Absolute GRBV coat protein (V1) quantification by PRM assay in leaf extracts. The intensities for each fragment *y*-ion, represented with different colors as indicated between the upper and lower bar graphs, were extracted by Skyline software from the raw data for each of the three analyzed peptides (sequences indicated on top), and the values from the technical replicates were summed to 1 value per plant extract. The log-transformed intensities are illustrated in each bar graph. *Upper panel: Leaf extracts L_D1 and L_H1 from three different plants, each, analyzed with 1 and 5 fmol heavy-labeled AQUA peptide spike-in.* The AQUA peptide concentration is indicated by the number after the plant replicate annotation given on the *x*-axis of the lower bar graph, e.g., L_D1_9115_1 for first replicate extract from GRBV-infected plant 9115 with 1 fmol AQUA peptide. *Lower panel: The second replicate leaf extracts L_D2 and L_H2 analyzed only with 1 fmol AQUA concentration.* The measured intensities of the native peptides are shown in the upper bar graphs in each panel, and the spiked-in AQUA peptide intensities in the lower bar graphs, respectively.

We first determined the linear range of transition intensities for the three C-terminally heavy isotope labeled AQUA peptides by spiking them into the combined petiole extracts from GRBV-free plants (Figure 7) by using the PRM approach as described under the section “Materials and Methods.” We found excellent linearity of the signal responses in a range between 0.1 and 50 fmol of peptides loaded on column. We subsequently used a concentration of 1 fmol AQUA peptide on column as an internal standard spike to all plant tissue extracts of plants 9106–9108 and 9115–9117, and 5 fmol with the first protein extraction replicate (Figure 1). As expected, we could not detect any traces of V1 peptides in any of the extracts of non-infected plants, corroborating the false positive match reported by MaxQuant for non-infected plant 9106 (Supplementary Figure 4). However, it was possible to quantify absolute amounts of all three GRBV V1 peptides in the petiole extracts of GRBV-infected plants with the exceptions of peptide I-K in the first sample extracts (P_D1) prepared from plants 9116 and 9117 at 1 fmol spike-in, and plant 9117 at 5 fmol spike-in (Figure 8). The absolute number of V1 molecules per milligram of wet tissue was calculated using the ratios calculated from the summed transition intensities of light and heavy peptides, the quantity of spiked-in peptides, the Avogadro constant of 6.022 × 10^23^ mol^−1^, and the weighed-in amount of tissue powder (Figure 9). We calculated in average of 2.37 × 10^8^, 1.73 × 10^8^, and 3.12 × 10^8^ V1 molecules/mg petiole tissue in plants 9115, 9116, and 9117, respectively. The calculated V1 protein concentrations differed between the two sample preparations P_D1 and P_D2, but also with peptide I-K (Figure 9 and Table 3). Peptide I-K gave consistently a lower response than the other two peptides as already seen in the calibration curve (Figure 7). The difference in calculated V1 concentration between the two petiole sample replicates can be explained by an over-estimated protein load with the protein extract P_D1 where protein yield was measured by BCA assay (Figures 1, 4). To our surprise, it was possible to detect traces of peptide AAFNIFQR in all L_D2 GRBV-infected leaf extracts (Figure 10). By using the absolute molecule numbers for peptide A-R in samples L_D2 and P_D2, the virus molecule load in leaves was calculated to be 5.9 times lower than in the petiole extracts. In terms of protein concentration, we could calculate an average of 0.068% (w/w) in petioles and 0.00021% (w/w) in the leaves. These comparisons between leaves and petioles are only based on the AAFNIFQR peptide and should therefore be treated with caution due to the detection of the native peptide at the lower limit of detection range.

**Table 3:** Light to heavy (L/H) peptide ratios, calculated GRBV V1 protein loads per 1 mg wet tissue, and relative protein concentration (% w/w) in GRBV-infected plant tissues.

				AAFNIFQR			NDVSGGGRNDVER			IYLSAASASGHTFK		
Tissue	Plant	Data-set	AQUA (fmol)	Ratio L/H mean ± SD	Number of V1 mol./1 mg tissue	% (w/w) V1	Ratio L/H mean ± SD	Number of V1 mol./1 mg tissue	% (w/w) V1	Ratio L/H mean ± SD	Number of V1 mol./1 mg tissue	% (w/w) V1
Petiole	9115	P_D1	5	0.041 ± 0.0024	4.80*E*+07	0.0038	0.024 ± 0.0021	2.80*E*+07	0.0022	0.006 ± 0.0004	7.43*E*+06	0.0006
	9116	P_D1	5	0.043 ± 0.0002	5.06*E*+07	0.0032	0.023 ± 0.0009	2.72*E*+07	0.0017	0.003 ± 0.0009	3.61*E*+06	0.0002
	9117	P_D1	5	0.038 ± 0.0014	4.58*E*+07	0.0035	0.011 ± 0.0019	1.37*E*+07	0.0011	n.d.		
	9115	P_D1	1	0.342 ± 0.0005	8.01*E*+07	0.0064	0.147 ± 0.0029	3.44*E*+07	0.0027	0.036 ± 0.0056	8.45*E*+06	0.0007
	9116	P_D1	1	0.134 ± 0.0037	3.17*E*+07	0.0020	0.126 ± 0.0073	2.98*E*+07	0.0019	n.d.		
	9117	P_D1	1	0.255 ± 0.0179	6.17*E*+07	0.0048	0.068 ± 0.0016	1.65*E*+07	0.0013	n.d.		
	9115	P_D2	1	8.636 ± 0.452	1.03*E*+09	0.098	6.826 ± 1.517	8.18*E*+08	0.078	0.600 ± 0.060	7.20*E*+07	0.0068
	9116	P_D2	1	5.307 ± 0.292	6.24*E*+08	0.028	4.239 ± 0.619	4.98*E*+08	0.023	1.002 ± 0.080	1.18*E*+08	0.0053
	9117	P_D2	1	9.660 ± 0.594	1.16*E*+09	0.078	5.790 ± 0.223	6.97*E*+08	0.047	1.554 ± 0.061	1.87*E*+08	0.0125
Leaf	9115	L_D2	1	0.059 ± 0.0013	2.75*E*+08	0.0004	n.d.			n.d.		
	9116	L_D2	1	0.035 ± 0.0063	1.62*E*+08	0.0002	n.d.			n.d.		
	9117	L_D2	1	0.009 ± 0.0012	4.03*E*+07	0.0001	n.d.			n.d.		
Petiole	mean	P_D1			0.53*E*+08	0.0039		0.25*E*+08	0.0018		0.07*E*+08	0.0005
Petiole	mean	P_D2			9.40*E*+08	0.0681		6.71*E*+08	0.0489		1.26*E*+08	0.0082
Leaf	mean	L_D2			1.59*E*+08	0.00021						

## Discussion

Despite its wide spread and representing a major threat to the wine industry in North America, GRBV virus particles have not been observed so far, nor have the potential protein products of the GRBV genome been detected in protein extracts of infected plants. Consequently, there is no serological assay available for a diagnostic test of GRBV infection. We initially set out to explore the possibility of developing such a test. In a first attempt, we screened the grapevine leaf proteome of several Gamay plants cultivated in a grapevine viral collection at Agroscope in Switzerland. We realized that protein yields were very low when using rather simple protein extraction protocols such as used for serological tests. Most tested protocols resulted in undefined smears with no discrete protein bands on SDS–PAGE. It became only possible to produce reproducible protein extracts with the described SDS extraction and the elaborate TCA/acetone precipitation and phenol wash protocols for leaf and petiole tissues, respectively, as described in the section “Materials and Methods.” Despite several washing steps of protein extracts, we observed that commonly used protein assays like BCA or Bradford gave un-reliable protein concentrations due to interfering, non-proteinaceous material still present in the samples (Figures 2, 4). The iBAQ protein intensities calculated by MaxQuant (Figure 4) corroborated the discrepancies between protein assay and effective column load. From these observations, we had to conclude that a reliable protein quantification from grapevine plant extracts was only possible after an additional protein clean-up step, for instance by way of SDS–PAGE protein fractionation. Indeed, column loads calculated based on SDS–PAGE staining intensities showed less variation between samples, increased iBAQ protein intensities, and numbers of identified proteins (Figure 4). Due to the variation in the data, we relied heavily on normalization for the discovery of proteins with a potential differential expression between GRBV-infected and non-infected plant tissue. Indeed, the coefficient of variation could be decreased in average from 101% to 77% or 70% between iBAQ and LFQ or iTop3 values, respectively (not shown).

Despite this uncertainty about equal column loads, we believe that the semi-quantitative proteome comparison produced meaningful data with the LFQ values delivering a better specificity and the iTop3 values probably a higher sensitivity. We therefore concentrated our efforts on using the iTop3 values. We called it iTop3 since we did impute missing intensity values on the peptide level, before summing the three most intense peptides to the protein abundance surrogate. Imputation of missing values is a double-edged sword. On one side it could give raise to false discoveries. On the other side, it strengthens statistical testing. Here we followed a published recommendation by Lazar et al. (2016), which we could confirm by our own simulations (not shown). When imputing missing peptide values with a left-censored strategy as done here prior to forming the top3 protein intensity, the normal distribution of log-transformed protein intensities is not disturbed, while this is clearly the case when doing so on the protein level. Moreover, we did impute peptides with caution, namely only when it had been quantified in at least two technical replicates from the same tissue type of the same plant.

We were able to identify grapevine proteins involved in the enzymatic cascade of flavonoid synthesis. Flavonoids are a group of secondary plant metabolites fulfilling a variety of functions, including coloring in order to attract pollinators or as a mechanism to fight against stress such as pathogen attacks. In the context of GRBV infection, the upregulation of the flavonoid biosynthetic pathway probably contributes to the leaf symptoms, the development of reddish color in GRBV-infected plants. Furthermore, *Grapevine leafroll-associated virus* infection of grapevine plants causes similar symptoms on vine leaves like GRBV. It has been shown earlier that *Grapevine leafroll-associated virus* infection of Merlot cultivars increased the transcription of genes involved in the flavonoid biosynthesis between 2- and 50-fold (Gutha et al., 2010). We can confirm the same plant response upon GRBV infection in Gamay plants on the proteome level in leaf and petiole tissues. Our results confirm other reports, where it was found that GRBV altered secondary metabolism as well as responses to stress (Blanco-Ulate et al., 2017).

Furthermore, we could confirm the presence of the GRSPaV by way of its 28 kDa coat protein in GRBV-free and infected plants at similar expression levels, confirming RT-PCR results produced at Agroscope (Reynard et al., 2018a).

We could also identify several proteins from the two bacteria *Novosphingobium* sp. *Rr 2-17* and *Methylobacterium* sp. *GXF4*. This was possible because the two bacteria have an association with grapevine, as their genomes were identified from bacteria isolated from parts of grapevine plants (Gan et al., 2012a,b). This association explains why protein sequences from these two bacteria were in the protein sequence database used for our peptide fragment spectra interpretation. *Methylobacteria* are a genus of *Rhizobia*, Gram-negative *Alphaproteobacteria*, which do live in symbiosis with plant roots where they fix nitrogen and are part of the leaf microbiota (Zarraonaindia et al., 2015). Gan and colleagues isolated *Methylobacterium sp. GXF4* from the xylem, indicating that this bacterium is part of the internal microbiota of grapevine plants too. Our findings do confirm this. *Novosphingobia* are a genus of *Sphingomonas*, also Gram-negative *Alphaproteobacteria*. *Novosphingobium Arabidopsis* was isolated from the roots of *Arabidopsis thaliana* (Lin et al., 2014), indicating that this type of bacteria could also have a symbiotic function. Furthermore, *Sphingomonas* have been recognized as a major species in the microbiome of Merlot leaves too (Zarraonaindia et al., 2015). Zarraonaindia et al. (2015) identified these bacteria through RNA sequencing. Our results do therefore confirm their existence at the proteome level. The microbiota of plants has received a lot of attention during recent years, as it may influence strongly the health of plants and as such mediates plant diversity and ecosystem function relationships (Müller et al., 2016; Laforest-Lapointe et al., 2017).

A further output of this study is that we could for the first time show the presence of GRBV virus in infected grapevine plants by identifying with a shotgun proteomics approach the two potential viral gene products V1 and V2 in petiole extracts, and absolutely quantify V1 in petioles and leaves. Total protein yields per milligram wet tissue were 20–40 times higher from leaf than petiole tissue. However, the leaf extracts were heavily dominated by RuBisCo proteins, with an average of 9.3–12.1% of the protein mass as calculated from iBAQ values, compared to 0.2–0.9% in petioles. Therefore, it is very likely that less GRBV protein was loaded onto the nano-LC system from leaf compared to petiole extracts, where no single protein was dominating the total protein mass like RuBisCo in leaves. This is corroborated by the observation that at a supposedly identical column load, it was possible to identify more protein groups in petiole extracts (Supplementary Table). In order to test the possibility that GRBV virus load is higher in petioles than in the total leaf tissue, we absolutely quantified the V1 protein by using heavily labeled standard peptides and a targeted nLC-MS/MS approach (PRM).

By sequence similarity to other *Geminiviridae* viruses, it is assumed that the V1 gene product represents the viral coat protein responsible for giving shape to virus particles, while the function of V2 protein is yet not clearly understood. We therefore quantified absolutely the V1 protein in order to get an idea about the virus load in grapevine petioles and leaves. The absolute V1 peptide quantification corroborated once more that the actual protein concentration in the grapevine tissue extracts were grossly over-estimated by the BCA protein assay. We determined an approximate 20-fold difference between spiked-in heavy labeled standard peptide and native non-labeled GRBV peptides between the SDS–PAGE- and BCA-based protein loads for the PRM runs represented by sample sets P_D1 and P_D2 (Figure 1 and Table 3). It is therefore not surprising that we could also quantify peptide A-R in the leaf extracts of sample set L_D2 with SDS–PAGE-based protein assay, but not in sample set L_D1 with BCA-based protein assay. Assuming that the SDS–PAGE-derived protein concentration is correct, we determined an average V1 protein load in the total protein extract of petioles of 0.042% with a standard deviation of 0.035%. The rather big deviation from the mean was due to differences in the three plants and also a somewhat inconsistent readback with peptide I-K compared to the two other standard peptides. We could extract about 20–40 times more protein per unit weight of wet tissue from leaves and found a 300 times lower V1 protein concentration in leaves compared to petioles, which indicates approximately a 10-fold higher absolute amount of V1 molecules in petioles. This factor is in good agreement with the ratio calculated from the average absolute numbers of V1 molecules of 5.9 calculated for peptide AAFNIFQR on sample replicates L_D2 and P_D2 (Table 3). Based on iBAQ, LFQ, and iTop3 intensities, it appeared that the V2 protein is present in higher copy numbers than V1. It is striking that intact GRBV virus particles could not have been visualized in infected plants, despite the fact that at least two viral gene products are expressed at a substantial molecule concentration in grapevine tissue. This apparent discrepancy opens a myriad of questions, e.g., how can the virus be transmitted by insects from one plant to another, or are commonly used protocols for virus enrichment and imaging too destructive for GRBV particles? Although a number of protocols have been developed for detecting plant viruses, very few of them make use of proteomics (Blouin et al., 2010). In this work, nLC-MS/MS was used to identify proteins from grapevine extracts. It was possible to detect two members of the grapevine virome. Therefore, proteomics might be a useful tool to detect plant viruses and could complement serological and molecular assays to aid plant virus diagnostic.

In summary, we present here for the first time the observation of *in situ* expression of two GRBV proteins in a range which exceeds clearly 100 million copies per 1 mg plant tissue for the coat protein and potentially even higher numbers for the viral sense 2 (V2) protein. GRBV infection triggers a defense mechanism in grapevine plants which involves the flavonoid metabolism. In addition, we found indications that the metabolic needs in terms of energy consumption are increased as a consequence, which might have a negative impact on grapevine berry yields and quality. GRBV infection appears to alter the cell wall rigidity of affected plants exemplified by increased yields of extracted proteins. We could also show that it was possible to identify members of the grapevine leaf and petiole microbiota using the hypothesis-free approach of shotgun proteome analysis. Last but not least, the direct comparison between leaf and petiole proteomics indicates that petioles are a potentially richer source for proteome studies than leaves, despite the recalcitrant property of the woody petioles.

## Author Contributions

NB carried out all sample preparation manipulations and was involved in finding suitable sample preparation protocols in the existing literature. SB-L ran the nLC-MS/MS, developed the PRM method, and interpreted the PRM data. A-CU was involved in the statistical data evaluation and manuscript writing. JB and J-SR cultivated the grapevine plants and collected the biological material. CD and J-SR designed the experiments and were involved in manuscript writing. MH was also involved in designing the experiments, drafted the manuscript, furthermore collected, interpreted, and evaluated all data, and designed figures and tables.

## Conflict of Interest Statement

The authors declare that the research was conducted in the absence of any commercial or financial relationships that could be construed as a potential conflict of interest.

## Footnotes

1 http://pantherdb.org/
