# Supplementary material for: Absolute Quantification of Grapevine Red Blotch Virus in Grapevine Leaf and Petiole Tissues by Proteomics
Source: Front Plant Sci. 2018 Nov 29;9:1735. doi: 10.3389/fpls.2018.01735 (PMC6281998; doi:10.3389/fpls.2018.01735)
Supplement: Supplementary file 1 [file Data_Sheet_1.pdf]

Absolute Quantification of *Grapevine Red Blotch Virus* in Grapevine Leaf and Petiole Tissues by Proteomics

Natasha Buchs[1], Sophie Braga-Lagache[1], Anne-Christine Uldry[1], Justine Brodard[2], Christophe Debonneville[3], Jean-Sébastien Reynard[2], Manfred Heller[1*]

[1]Proteomics and Mass Spectrometry Core Facility, Department for BioMedical Research (DBMR), University of Bern, CH-3010 Bern, Switzerland
[2]Institute for Plant Production Science, Agroscope, CH-1260 Nyon, Switzerland
[3]Bioreba AG, CH-4153 Reinach, Switzerland

*** Correspondence:**
Dr. Manfred Heller
manfred.heller@dbmr.unibe.ch

Supplementary Figures

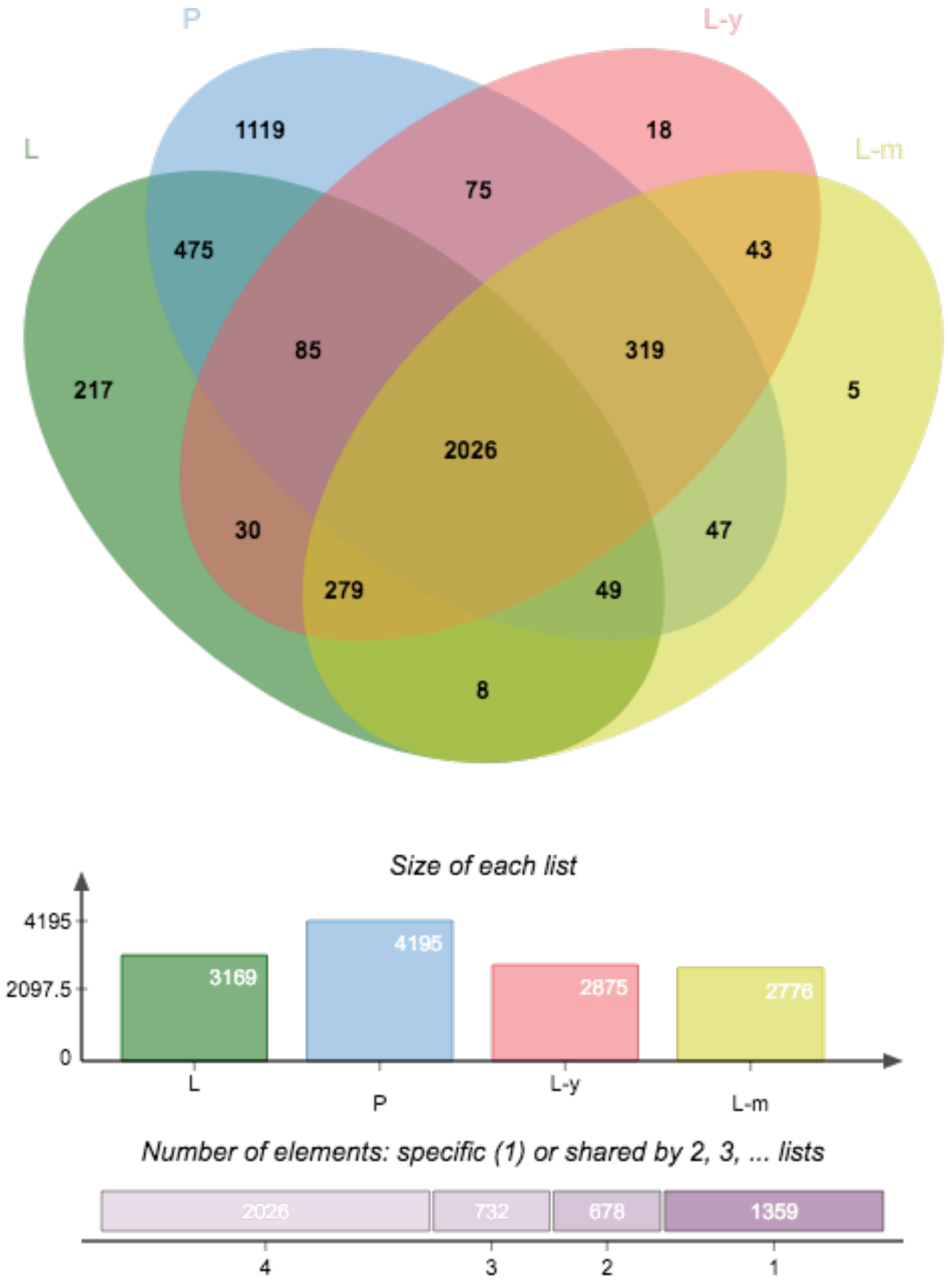

Supplementary Fig. 1: Venn diagram comparing protein groups identified. L (green) stands for the two leaf replicate analyses using pooled leaves from three different plants L_H1, L_H2, L_D1, and L_D2; P (blue) the same plants but using petioles P_H1, P_H2, P_D1 and P_D2; L-y (red) are the set of 10 young leaves L_yH and L_yD; and L-m (yellow) are from the mature leaves L_mH and L_mD, respectively. The numbers indicate protein groups quantified by iTop3

approach. The diagram was produced with the jvenn tool: http://jvenn.toulouse.inra.fr/app/example.html

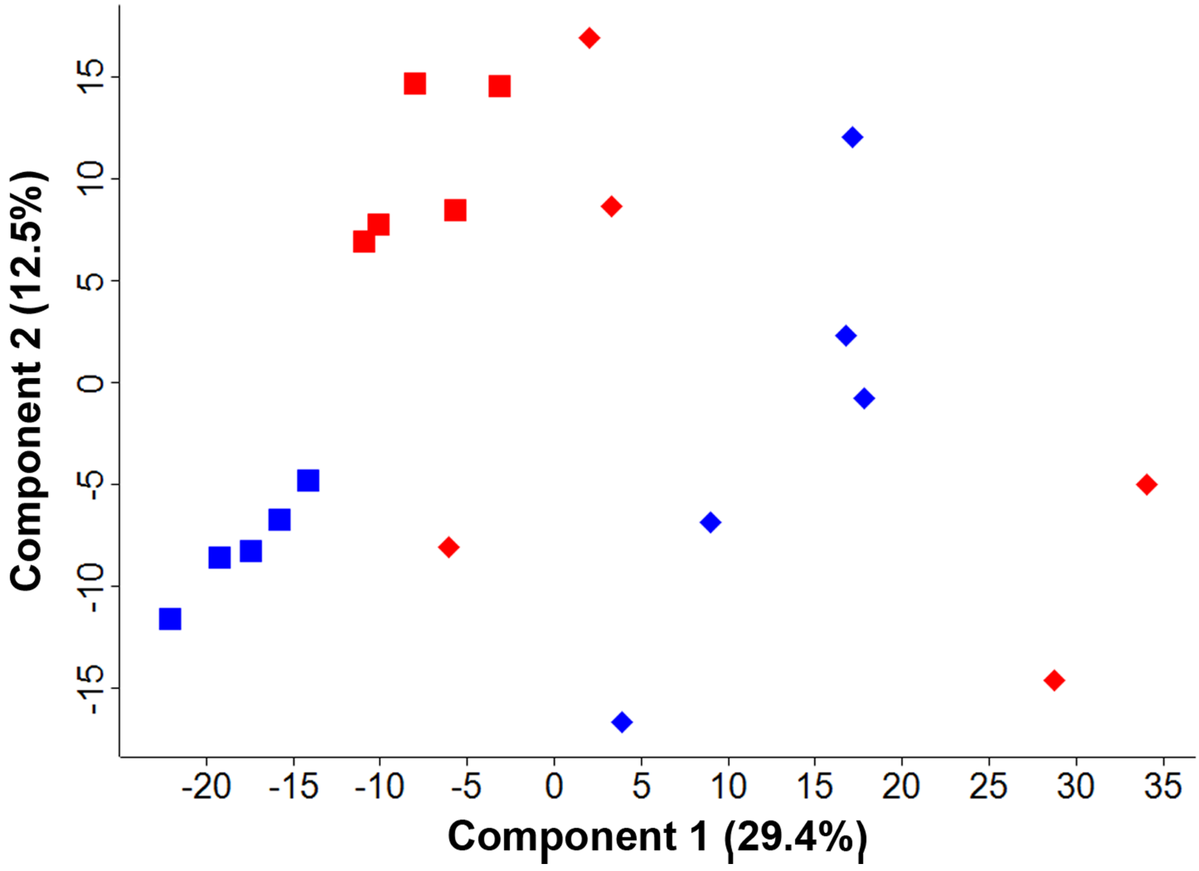

Supplementary Fig. 2: Principal component analysis of iTop3 protein group intensities quantified in all 20 leaves of plants 9021 (non-infected) and 9034 (GRBV-infected). The 1483 protein groups quantified in all 20 leaves were submitted to a principal component analysis. The first two main components were used for this plot, with the contribution to the difference between leaves given in a per-cent scale on the axes. The diamonds represent young and the squares mature leaves, respectively. The symbols in blue color represent the leaves from the non-infected plant 9021, while the red colored symbols are the ones from the GRBV-infected leaves.

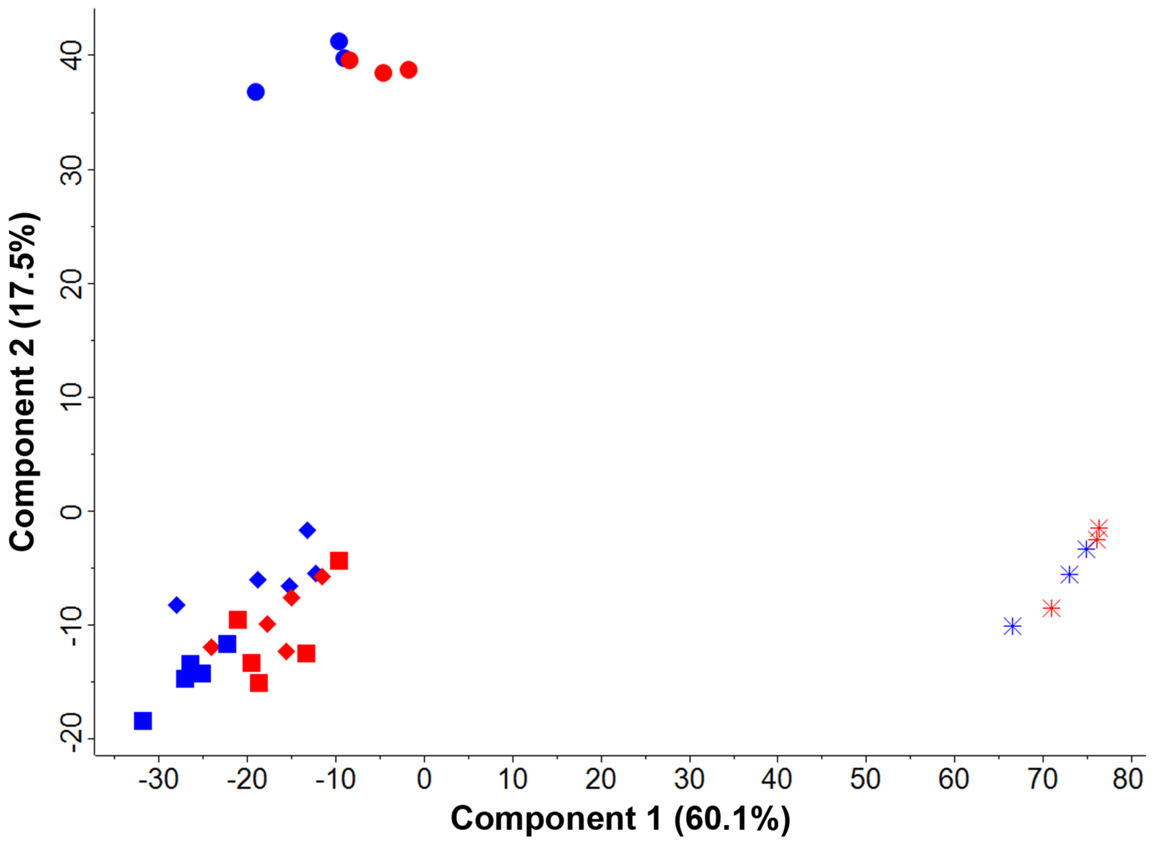

Supplementary Fig. 3: Principal component analysis of iTop3 protein group intensities quantified in all 32 tissue extracts. There were 1223 protein groups quantified in all 44 tissue extracts and submitted to a principal component analysis. The first two main components were used for this plot, with the contribution of each component given in a per-cent scale on the axes. The diamonds represent young (L-yH, L_yD), the squares mature (L_mH, L_mD) leaves from plants 9021 and 9034, the circles leaves, and the stars petioles from plants 9106-9108 and 9115-9117, respectively. The symbols in blue color represent the tissue from the non-infected, while the red colored symbols are the ones from the GRBV-infected plants.

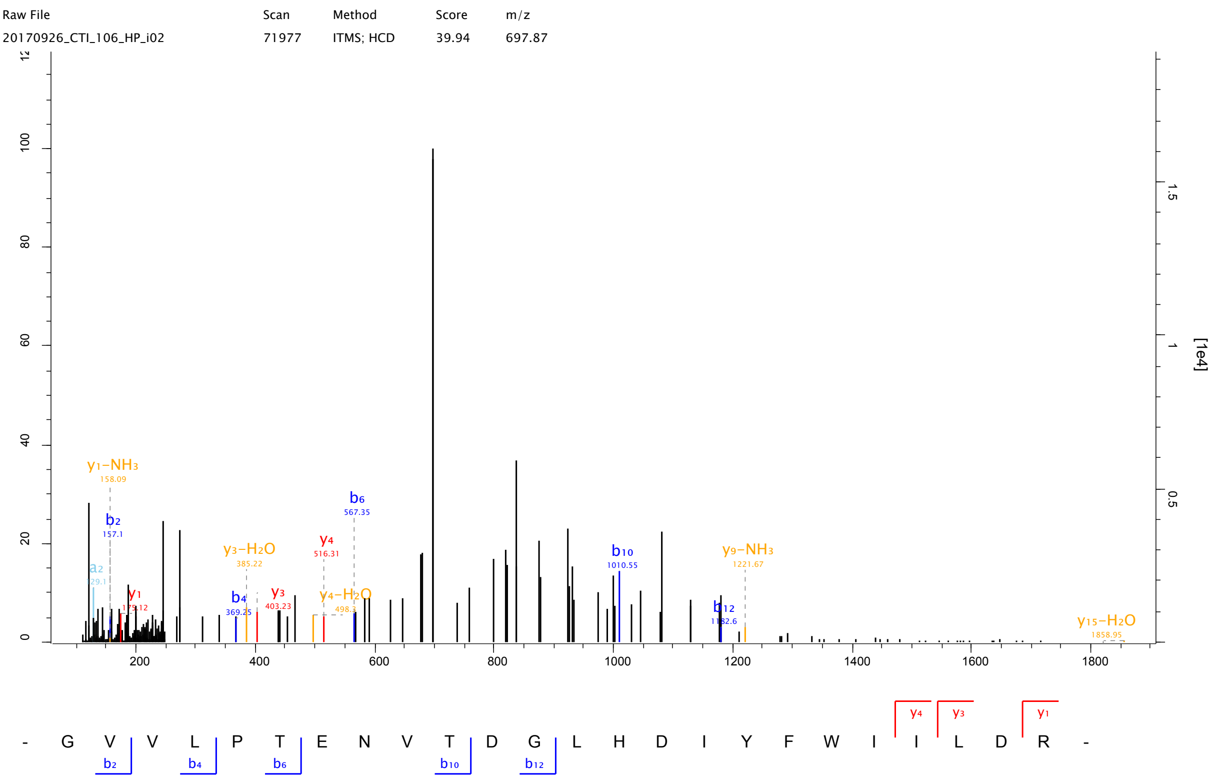

Supplementary Fig. 4: False positive GRBV V1 peptide identification. This unique peptide spectrum match for peptide GVVLPTENVTDGLHDIYFWIILDR, identified only in one nLC-MS/MS run of the petiole extract of non-infected plant 9106, was reported by MaxQuant/Andromeda software. This identification must be rejected as a random match, due to the weak evidence on low intense fragment peaks matching non-consecutive b and y ions. The false positive nature of this identification is corroborated by the fact, that the targeted analysis with heavy labeled internal V1 peptide standards did not detect any traces of V1 peptides in a total of 7 replicate PRM runs of non-infected petiole tissue extracts.

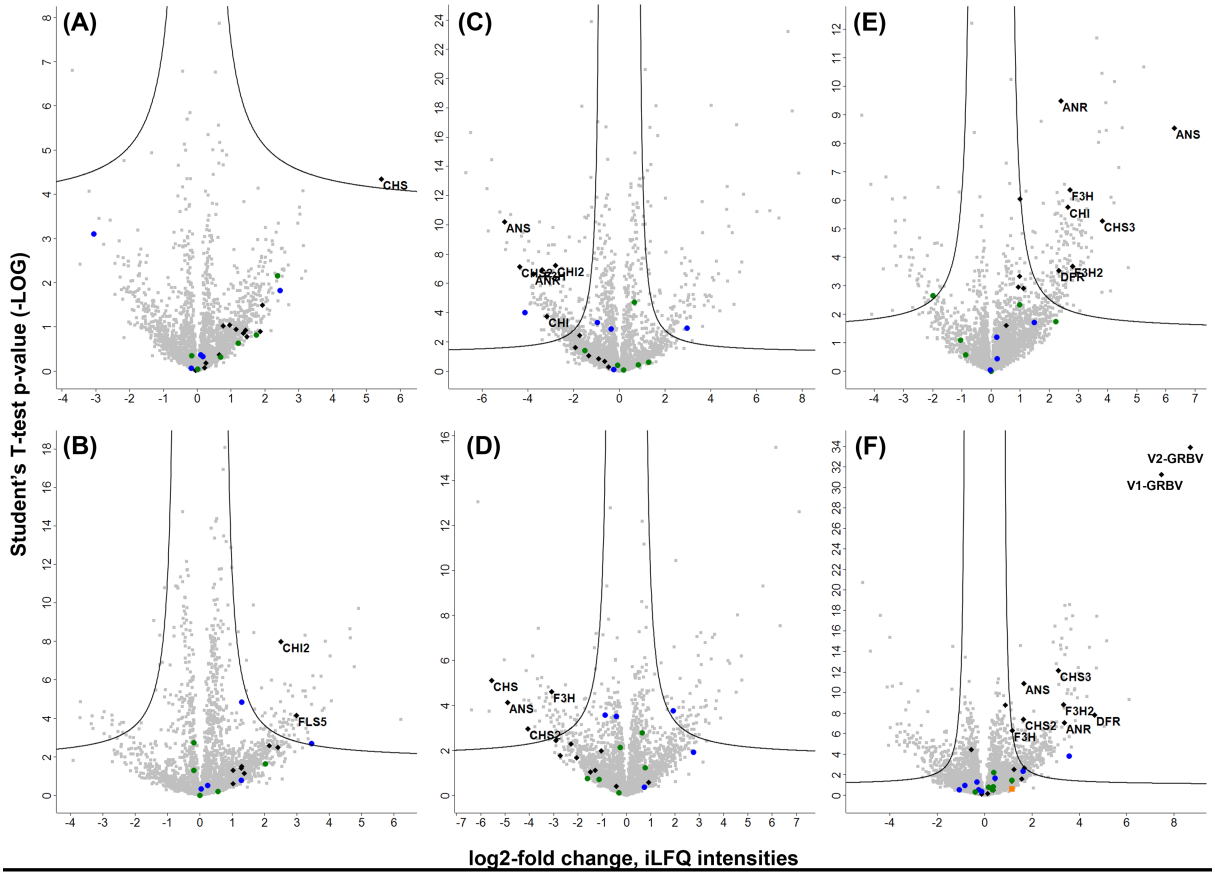

Fig. 5: Volcano plots indicating genes involved in flavonoid biosynthesis. Student's t test p-values (as -LOG) from the different tests performed are given on the y-axis with the corresponding LOG2-fold changes of LFQ intensities on the x-axis, respectively. Black diamonds represent flavonoid biosynthesis enzymes and the two GRBV proteins, only identified in petioles. The gene names of differentially expressed proteins are given. The ORF5 protein of GRPaV also quantified only in petioles is indicated by an orange square in panel F. The filled circles represent the two identified bacterial species, Methylobacterium in blue and Novosphingobium in green. The following test results are shown in the six panels: A = GRBV-infected (L_yD) *vs* non-infected young leaves (L_yH), B = GRBV-infected (L_mD) vs non-infected mature leaves (L_mH) , C = mature (L_mH) against young non-infected leaves (L_yH) from the same plant, D = mature (L_mD) against young GRBV-infected leaves (L_yD) from the same plant, E = GRBV-infected (L_D1 & L_D2) against non-infected leaves (L_H1 & L_H2) from three different plants, and F = GRBV-infected (P_D1 & P_D2) against non-infected petioles (P_H1 & PH2) from three different plants. If the LOG2-fold change is on the positive side, the corresponding protein was more abundant in the first mentioned sample set, if negative it was more abundant in the second sample set, respectively. Only two proteins were significantly changed in expression between non-infected and GRBV-infected young leaves, with chalcone synthase (CHS) being upregulated in latter (panel A). In mature leaves of the same plants, 63.6% of proteins had a higher expression level in GRBV-infected plants and 69 reached our set significance level compared to only 21 with an increased expression in healthy tissue (panel B). This trend was confirmed by the analysis of leaves from different plants, where 62.5% of all proteins showed an increased expression level in GRBV-infected leaves with 164 being significant, compared to only 49 significantly increased proteins in healthy plants (panel E). In petioles, we also detected more proteins with a positive test difference between GRBV-infected and non-infected plants (54.1%) and 233 reaching significance compared to 115 of significant lower expression (Fig. 5, panel F).
